# Generalized Gamma-CUSUM control chart with application of COVID-19 deaths

**DOI:** 10.1371/journal.pone.0281360

**Published:** 2023-02-02

**Authors:** Olatunde Adebayo Adeoti, Kayode Samuel Adekeye

**Affiliations:** 1 Department of Statistics, School of Physical Sciences, Federal University of Technology Akure, Akure, Nigeria; 2 Faculty of Natural Sciences, Department of Mathematical Sciences, Redeemers University Ede, Ede, Nigeria; Bucharest University of Economic Studies: Academia de Studii Economice din Bucuresti, ROMANIA

## Abstract

The increase in the number of infections and the worrisome state of mortality linked to the COVID-19 pandemic demand an optimal statistical model and efficient monitoring scheme to analyze the deaths. This paper aims to model the COVID-19 mortality in Nigeria using four non-normal distributions grouped under the generalized gamma distribution, by specifying the best-fit distribution to model the number of deaths linked to the COVID-19 pandemic. In addition, a control chart to monitor the COVID-19 deaths based on the best-fit distribution is proposed. The performance of the proposed Gamma-CUSUM chart as a monitoring scheme was compared with the standard normal-CUSUM chart. The results revealed that the Gamma-CUSUM chart first signals a change in the number of deaths on day 68 while there was no change in the number of deaths for the standard normal-CUSUM chart. Also, the exact point of change was visible on the Gamma-CUSUM chart which was impossible on a standard normal-CUSUM control chart.

## Introduction

The novel coronavirus SAR-CoV-2 virus (also known as COVID-19) [1] is a highly contagious disease first detected in Wuhan, China in December 2019 [2]. It is one of the rare diseases that has constituted the biggest threat to the existence of humanity and has negatively impacted the economies of many countries. The virus spreads primarily from person to person through respiratory droplets [3]. Some of the symptoms of the disease may include fever, cough shortness of breath, chills, muscle pain, and loss of taste or smell which may appear 2–14 days after exposure [1,4]. The infection rate and the number of deaths due to the COVID-19 pandemic across the globe are high with a new strain of the virus discovered recently. However, to mitigate the effect of the pandemic, considerable research effort has led to the development of vaccines that have been approved for use in reducing the impact of COVID-19 infections and deaths.

To date, COVID-19 cases have been confirmed in over 188 countries on different continents, Africa and including Nigeria [5]. Globally, the number of confirmed cases as of 7^th^ October 2021 is 236 132 082 and the number of deaths due to COVID-19 is given as 4 822 472. In Nigeria, the first index case of COVID -19 was detected on 27^th^ February 2020 [6]. Since the first index case in Nigeria, there has been a gradual rise in the number of confirmed cases, recoveries, and mortality. Daily records of confirmed cases, recoveries, and deaths including cumulative figures are provided by Nigeria Centre for Disease Control (NCDC). As of 7^th^ October 2021, the total number of confirmed COVID-19 cases, discharged cases, active cases, and deaths in Nigeria as given by NCDC are 206920, 194651, 9471, and 2740 respectively [6].

To detect the presence of a virus, tests are conducted on individuals; an infected person is isolated and quarantined to prevent the spread of the virus. However, there is apprehension about the total number of confirmed cases in Nigeria due to low testing. Recently, there has been an improvement in the number of COVID-19 testing centers and an increase in testing capacity across the states in Nigeria due to the support of international donor agencies and the Federal government. Also, the government has given non-pharmaceutical guidelines to mitigate the spread of the virus through social distancing rules, isolation and quarantine, use of nose masks, hand washing, and smaller gathering of people at a time. This had a positive effect in controlling the outbreak, but, with a substantial loss in economic and social costs during the lockdown [7]. Yet, these measures have not helped in preventing deaths among the infected and vulnerable people.

Since the outbreak, many studies have been undertaken to estimate the growth rates and understand the transmission dynamics of COVID-19. Zhao et al. [8] estimated the growth rate of COVID-19 infection in China using the exponential growth model. Kucharski et al. [9] investigated the transmission dynamics of COVID-19 infection using a mathematical model to assess the effectiveness of several control measures. Chen et al. [10] proposed a mathematical model for estimating the transmissibility rate of the coronavirus and showing a higher transmissibility rate for COVID-19 than for some other viruses. Statistical analysis and monitoring of COVID-19 data were also explored by some researchers using sampling plans under neutrosophic statistics. Aslam et al. [11] proposed a gamma control chart based on generalized multiple dependent states using the COVID-19 mortality data. Sherwani et al. [12,13] examined the performance of the sign and Kruskal Wallis tests under indeterminacy with applications to the COVID-19 reproduction rate and COVID-positive daily occupancy in ICU. Rao et al. [14] proposed a time-truncated sampling plan using COVID-19 data for Weibull distribution under indeterminacy. For more details regarding different studies on the COVID- 19 pandemic, interested readers are referred to the work of [15–21].

Several control charts have been proposed in the literature for the detection of an abnormal process. These control charts are used for detecting large and small-to-moderate shifts in the process variable. Notable among the control charts include the Shewhart chart [22], classical exponentially weighted moving average (EWMA) chart [23], and cumulative sum (CUSUM) chart [24]. While the Shewhart chart is efficient in detecting large shifts, the classical EWMA and CUSUM charts detect small-to-moderate shifts efficiently. New monitoring schemes such as HWMA charts, mixed control charts, and progressive mean charts which are extensions and modifications of the EWMA and CUSUM charts have been studied in the literature, but the efficiency of such monitoring schemes has been criticized by Knoth et al. [25].

Usually, the assumption in the monitoring of process shift is that the statistical distribution of the process variable follows the normal distribution. However, this is not the case in practice, because statistical distribution may follow some non-normal distributions. Hence, monitoring the COVID-19 deaths using control charts to detect abnormal/unnatural variation required identifying an appropriate statistical distribution to analyze the number of COVID-19 deaths. It should be noted that analysis of COVID-19 data is a good indicator and a veritable means of detecting the worrisome state of the effect of the virus in Nigeria and across the globe.

The generalized gamma distribution has been studied by several authors including Agarwal and Al-Saleh [26] who applied generalized gamma to study hazard rates. Nadarajah and Gupta [27] introduced generalized gamma distribution with application to fitting drought data. Cordeiro et al. [28] studied generalized gamma distribution using an exponentiated method and applied it to lifetime and survival analysis. The number of deaths is the focus of this paper based on the fact that death is a factor that eliminates lives and thus challenges the attainment of one of the sustainable development goals (SDGs). Therefore, an appropriate statistical model for the COVID-19 data based on the generalized gamma distribution is desirable for identifying the best-fit parametric model for monitoring the number of deaths.

Hence, this study aims to model the distribution of the number of deaths due to COVID-19 in identifying the appropriate (best-fit) parametric model using generalized gamma distribution to discover important patterns in COVID-19 data over the period under consideration which will enable the Federal government to have firsthand information on curtailing the pandemic in Nigeria. The best-fit mortality distribution modeled will reflect mortality from the different age groups of infected persons in the different states. As a follow-up, it monitors the COVID-19 death for abnormal patterns using the CUSUM chart based on the best-fit distribution, unlike the standard CUSUM which assumes that process data is normally distributed. This will prevent false conclusions to be made using the standard (normal) CUSUM chart. In the subsequent sections, the probability density function (PDF), the cumulative distribution function (CDF) of the generalized Gamma distribution, the description of the COVID-19 data, the procedure for obtaining the best-fit distribution, the design of the Gamma-CUSUM chart, and application to COVID-19 is discussed.

## Generalized Gamma Distribution (GGD)

The three-parameter generalized Gamma distribution first introduced by Stacy [29] is a generalization of the two-parameter gamma distribution. It is used to determine which parametric model is appropriate for a set of data. The GGD under consideration is an extremely flexible distribution for data modeling and has respectively PDF and CDF of the form

$$f\left(x,\;\lambda ,\;\alpha ,\;\beta \right)=\frac{\beta }{\lambda^{\alpha \beta }\Gamma \left(\alpha \right)}x^{\alpha \beta -1}e^{-{\left(x/\lambda \right)}^{\beta }}\;\;\lambda ,\;\alpha ,\;\beta >0,$$ (1)

and

Fx,λ,α,β=∫0xfu,λ,α,βdu=γαβ,xλβΓαβλ,α,β>0 (2)

where *λ* is the scale parameter, *α* and *β* are the shape parameters and *γ*(.) is the incomplete gamma function. The distribution has the exponential, Weibull, Gamma, and lognormal distributions as special cases and it is often used to identify which parametric model fits a given set of data. The distribution in Eq (1) becomes the exponential distribution if *α* = *β* = 1, gamma distribution if *β* = 1, Weibull distribution if *α* = 1, and lognormal distribution if *α* → ∞.

## The data

The data used in this study was obtained from Nigeria Center for Disease and Control (NCDC) website (https://ncdc.gov.org). The data consists of daily reported COVID-19 confirmed cases, recoveries, active cases, and the number of deaths between 11^th^ April and 7^th^ September 2020. As of 7^th^ September 2020, the cumulative number of COVID-19 confirmed cases, active cases and COVID-19 deaths are 55160, 10868, and 1061 respectively. Our interest in this paper is the daily reported COVID-19 number of deaths between the periods of study. Exploratory analysis and graphical tests of normality were carried out on the reported number of COVID-19 deaths. Also, the best-fit distribution for COVID-19 data was determined using the generalized gamma distribution, and monitoring the COVID-19 number of death was done using the CUSUM control chart.

**Fig 1:**
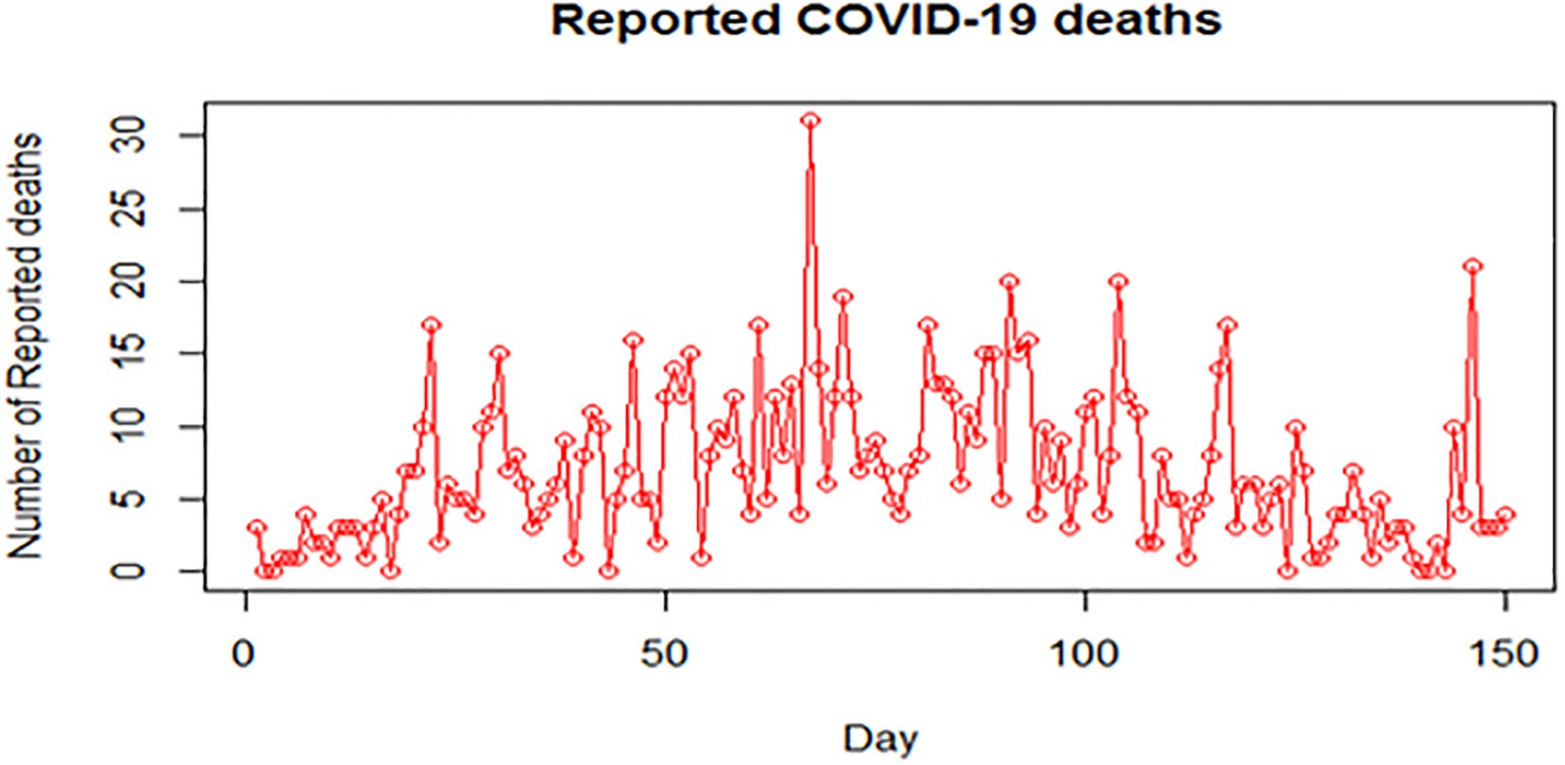
Daily reported COVID-19 number of deaths in Nigeria (11^th^ April -7^th^ September 2020).

**Fig 2:**
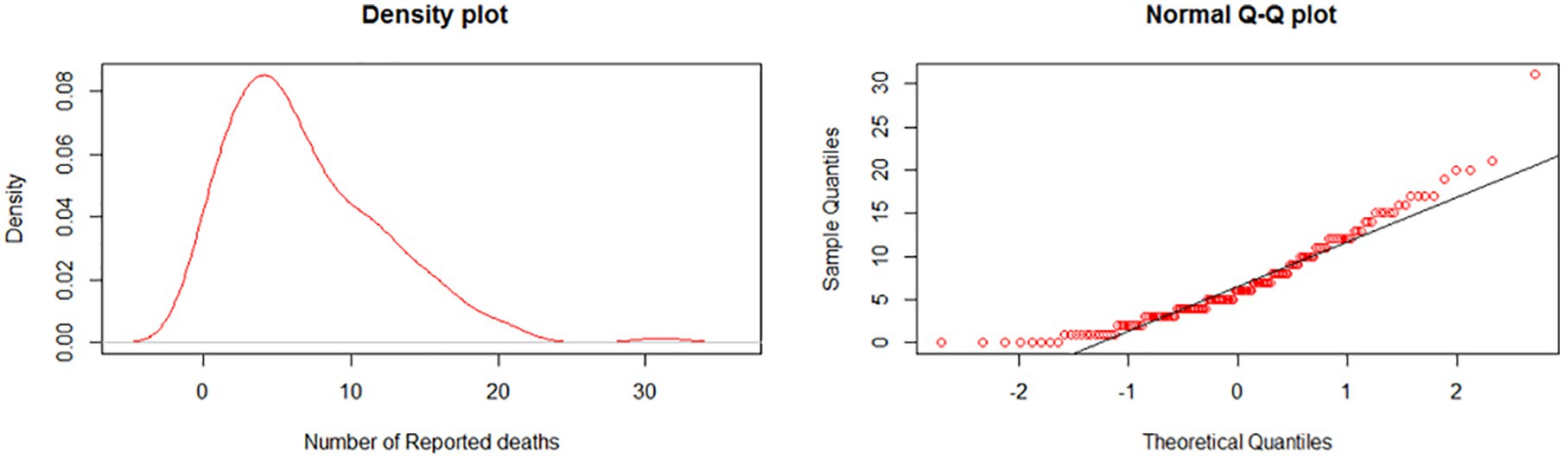
Density and normal QQ plot of the COVID-19 number of deaths.

The exploratory analysis of the reported COVID-19 deaths shows that the coefficient of skewness is 1.1471, the coefficient of Kurtosis is 4.908, and the mean value was greater than the median value. This implies that the data is positively skewed. A plot of the COVID-19 number of deaths in Nigeria within the period of study was presented in Fig 1 and a further graphical presentation using a QQ plot and density plot is presented in Fig 2.

The plots in Fig 2 further confirm that the distribution of the data is positively skewed and heavily tailed. Thus, a CUSUM chart based on the normal distribution will not be appropriate to study the data. Hence, there is a need to search for the appropriate model of distribution for the data.

## Best-fit distribution for the data

The COVID-19 death is a count data recorded over a time interval. Here, the distribution of COVID-19 number of deaths is modeled in this paper with the generalized gamma distribution (GGD) to obtain the best-fit distribution which is important in the design of the CUSUM control chart. The mortality distribution modeled here will reflect mortality from the different age brackets of infected cases in the different states and regions. The model can approximate the dynamics of COVID-19 and discover important patterns in the data. The generalized gamma distribution which encompasses the exponential, gamma, and Weibull distribution presents a flexible family in the varieties of shapes and hazard function which make it suitable for modeling real-life data to determine which parametric model is appropriate for the COVID-19 data. The distribution of the number of deaths due to COVID-19 is modeled with the gamma distribution for convenience where within the range of observation (data) we consider whether or not discretized continuous approximation fits the data since the data is an aggregate of deaths in the region where sparse data or no data on death due to COVID-19 are recorded.

To fit the best distribution to the data, we used the *fitdist* function in the *fitdistrplus* package in the R language developed by [30] and fit the GGD on the COVID -19 number of deaths. The parameters of the four generalized gamma distributions were estimated using the maximum likelihood estimation (MLE) method to obtain the model that best described the data. Table 1 gives the goodness-of-fit statistics and criteria for the four flexible distributions.

**Table 1:** Goodness-of-fit statistics and criteria.

	Weibull	Gamma	Lognormal	Exponential
**Kolmogorov-Smirnov statistic**	0.07798792	0.08848669	0.06545736	0.1845851
**Cramer-von Mises statistic**	0.09701833	0.20831520	0.08982366	0.9628920
**Anderson-Darling statistic**	0.67377891	1.73021666	0.70854618	5.7583306
**Akaike’s Information Criterion (AIC)**	826.9615	825.7383	835.2295	855.2839
**Bayesian Information Criterion (BIC)**	832.8732	831.6499	841.1411	858.2397
**p-value**	0.1731	0.0145	0.00000014	0.0297

Using the exponential distribution model, the parameters of the distribution were determined to be 0.13 with a standard error of 0.011 for the rate parameter. The log-likelihood, Akaike Information Criterion (AIC), and Bayesian Information Criterion (BIC) for the model are -426.64, 855.28, and 858.24, respectively. For the gamma distribution model, the parameters of the distribution were determined to be 1.97 with a standard error of 0.217 for the shape parameter and 0.26 with a standard error of 0.03 for the rate parameter. The log-likelihood, AIC, and BIC for the model are -410.87, 825.74, and 831.65, respectively. Furthermore, using the Weibull distribution model, the parameters of the distribution were determined to be 1.48 with a standard error of 0.096 for the shape parameter and 8.23 with a standard error of 0.492 for the scale parameter. The log-likelihood, AIC, and BIC for the model are -411.48, 826.96, and 832.87, respectively. Finally, using the Lognormal distribution model, the parameters of the distribution were determined to be 1.72 with a standard error of 0.07 for the scale parameter and 0.80 with a standard error of 0.048 for the shape parameter. The log-likelihood, AIC, and BIC for the model are -415.61, 835.23, and 841.14 respectively.

From the above results, it is clear that the gamma distribution best describes statistically the distribution of the number of deaths due to COVID-19 in this study. Therefore, we developed a Gamma-CUSUM control chart to investigate the COVID-19 deaths in Nigeria.

## Design of generalized Gamma-CUSUM control chart

The proposed CUSUM chart for monitoring the COVID-19 number of deaths is designed based on the gamma distribution which is the best-fit distribution for the COVID-19 number of deaths in Nigeria. We monitor upward shifts in the COVID-19 number of deaths and proposed a one-sided Gamma CUSUM control chart. The upper Gamma-CUSUM statistic is defined by

$$C_{n}^{+}=\text{max}\left(0,\;C_{n-1}^{+}+\left(X_{i}-K\right)\right)$$ (3)

where *X*_*i*_ represents the number of deaths that follow the gamma distribution, $C_{n}^{+}$ is the gamma CUSUM score for some case *n*, *K* is the reference value and $C_{0}^{+}$ is the non-negative head-start given as $C_{0}^{+}=\theta_{0}$. When $C_{n}^{+}>H$, the system signals an out-of-control condition, indicating that the COVID-19 number of deaths has exceeded the control limit. In designing the Gamma-CUSUM chart, the choice of *K* and *H* are fundamental in the application. The reference value *K* is obtained as a log-likelihood ratio given by (cf. [31])

$$K=\frac{\gamma \left(ln\theta_{1}-\theta_{0}\right)}{\theta_{0}^{-1}-\theta_{1}^{-1}},$$ (4)

where *γ* is a fixed shape parameter, *θ*_0_ is an in-control scale parameter if known or ${\hat{\theta }}_{0}$ if it is estimated and *θ*_1_ is an out-of-control scale parameter and

$$H=h\theta_{0}$$ (5)

is the decision limit which depends on the shape parameter *γ*, the ratio θ1θ0 and it is chosen to give a pre-specified in-control average run length (IC ARL) performance. The ARL is the expected number of samples to signal an abnormal condition which is one of the most commonly used measures of evaluating the performance of a control chart. A good control chart is expected to have a large ARL value for an in-control process. The ARL performance of the control chart for non-normal distributions has been studied by many researchers including Varderman and Ray [32] that derived the exact ARL value for the exponential distribution. Acosta-Mejia et al [33] assessed the performance of a CUSUM chart using ARL under chi-square distribution. The theoretical analysis of the ARL of the Gamma-CUSUM chart was studied by Huang et al. [31] who evaluated the run-length distribution of the CUSUM chart for monitoring changes in the scale parameter under gamma distribution based on the piecewise collocation method.

## Application to COVID-19 data

For monitoring the COVID-19 number of deaths in Nigeria using the control chart, the CUSUM chart with the best-fit distribution is applied. Though the CUSUM chart is usually based on the assumption that the quality characteristic follows the normal distribution, however, in this study, using the daily reported COVID-19 number of deaths within the period of study, the shape parameter of the gamma distribution was estimated to be 1.9669 and rate parameter is 0.2650. Thus, the in-control scale parameter is estimated as *θ*_0_ = 3.77365.

Suppose our objective is to detect a 25% increase in the COVID-19 number of deaths (note that percentage values less than 25% could also be considered in detecting an increase in the number of deaths), then the new scale parameter will be estimated to be *θ*_1_ = 4.71706. Thus, θ1θ0=1.25. Therefore, the reference value using Eq (3) will be *K* = 8.281377.

The approximate threshold, *h* for IC ARL value of 370 obtained from [31] given that the shape parameter *γ* ≈ 2 is determined as 13.2. Hence, the decision limit for the Gamma-CUSUM chart using Eq (5) is given as

$$H=h\theta_{0}=49.81218.$$

**Fig 3:**
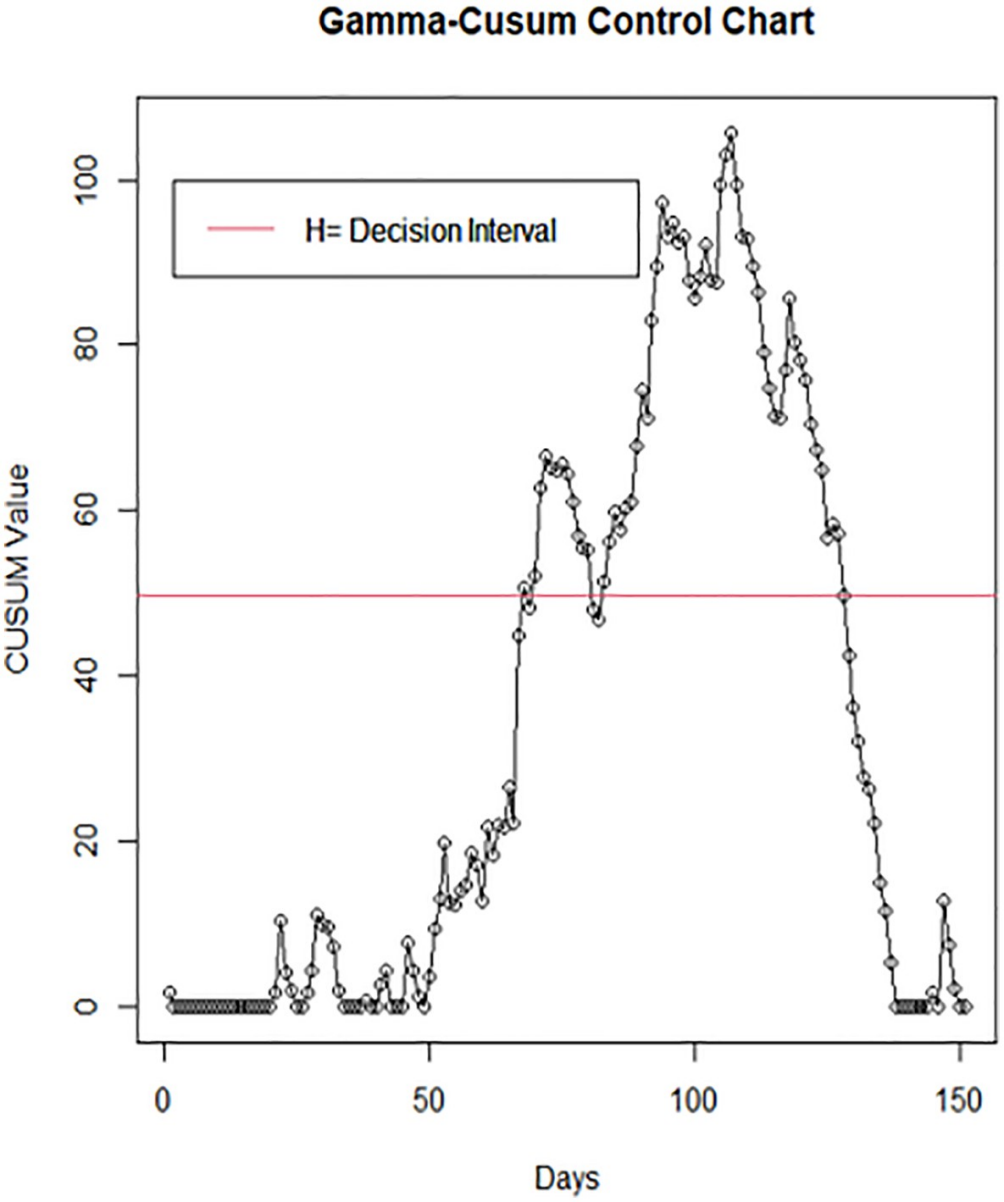
Gamma-CUSUM chart of COVID-19 number of deaths in Nigeria.

Therefore, the CUSUM statistic $C_{n}^{+}$ signals an out-of-control at the first *n* for which $C_{n}^{+}>49.81$. The summary of the upper Gamma-CUSUM statistic $C_{n}^{+}$ for the COVID-19 number of deaths is presented in Table 2. A plot of the statistic in Table 2 produced the Gamma-CUSUM control chart presented in Fig 3.

**Table 2:** Upper Gamma-CUSUM statistic for COVID-19 number of deaths.

Day	Number of Deaths	Upper CUSUM Statistic, $C_{n}^{+}$	Day	Number of Deaths	Upper CUSUM Statistic $C_{n}^{+}$	Day	Number of Deaths	Upper CUSUM Statistic $C_{n}^{+}$
1	3	1.6391	52	12	13.1559	103	4	87.8056
2	0	0	53	15	19.8745	104	8	87.5748
3	0	0	54	1	12.5931	105	20	99.2934
4	1	0	55	8	12.3117	106	12	103.0121
5	1	0	56	10	14.0304	107	11	105.7307
6	1	0	57	9	14.7489	108	2	99.4493
7	4	0	58	12	18.4676	109	2	93.1679
8	2	0	59	7	17.1862	110	8	92.8866
9	2	0	60	4	12.9049	111	5	89.6052
10	1	0	61	17	21.6235	112	5	86.3238
11	3	0	62	5	18.3421	113	1	79.0424
12	3	0	63	12	22.0608	114	4	74.7610
13	3	0	64	8	21.7794	115	5	71.4797
14	1	0	65	13	26.4980	116	8	71.1983
15	3	0	66	4	22.2167	117	14	76.9169
16	5	0	67	31	44.9353	118	17	85.6355
17	0	0	68	14	50.6539	119	3	80.3542
18	4	0	69	6	48.3725	120	6	78.0728
19	7	0	70	12	52.0912	121	6	75.7914
20	7	0	71	19	62.8098	122	3	70.5100
21	10	1.7186	72	12	66.5284	123	5	67.2287
22	17	10.4372	73	7	65.2470	124	6	64.9473
23	2	4.1559	74	8	64.9657	125	0	56.6659
24	6	1.8745	75	9	65.6843	126	10	58.3845
25	5	0	76	7	64.4029	127	7	57.1032
26	5	0	77	5	61.1215	128	1	49.8218
27	4	1.7186	78	4	56.8401	129	1	42.5404
28	10	4.4372	79	7	55.5588	130	2	36.2590
29	11	11.1559	80	8	55.2774	131	4	31.9777
30	15	9.8745	81	1	47.9960	132	4	27.6963
31	7	9.5931	82	**7**	46.7146	133	7	26.4149
32	8	7.3117	83	13	51.4333	134	4	22.1335
33	6	2.0304	84	13	56.1519	135	1	14.8521
34	3	0	85	12	59.8705	136	5	11.5708
35	4	0	86	6	57.5891	137	2	5.2894
36	5	0	87	11	60.3078	138	3	0.0080
37	6	0	88	9	61.0264	139	3	0
38	9	0.7186	89	15	67.7450	140	1	0
39	1	0	90	15	74.4636	141	0	0
40	8	0	91	5	71.1822	142	0	0
41	11	2.7186	92	20	82.9009	143	2	0
42	10	4.4372	93	15	89.6195	144	0	0
43	0	0	94	16	97.3381	145	10	1.7186
44	5	0	95	4	93.0567	146	4	0
45	7	0	96	10	94.7754	147	21	12.7186
46	16	7.7186	97	6	92.4940	148	3	7.4372
47	5	4.4372	98	9	93.2126	149	3	2.1559
48	5	1.1559	99	3	87.9313	150	3	0
49	2	0	100	6	85.6498	151	4	0
50	12	3.7186	101	11	88.3684			
51	14	9.4372	102	12	92.0869			

From Fig 3, it can be observed that the control chart first signals an out-of-control on day 68. The Figure also reveals that the COVID-19 number of deaths has been on a consistent rise cumulatively from day 68 to day 128 followed by a gradual decline for the remaining days. This study has established that the COVID-19 number of deaths in Nigeria remained in out-of-control for the next 60 days. Hence, the reasons for the consistent rise in the COVID-19 number of deaths within this period in Nigeria and the gradual decline thereafter required further investigation. The period 17^th^ June to 15^th^ August is regarded as the rainy season where the weather is very cold. Though there is no scientific evidence of a correlation between the number of infections/deaths and weather conditions, further research can be considered in temperate regions to verify this observation.

**Fig 4:**
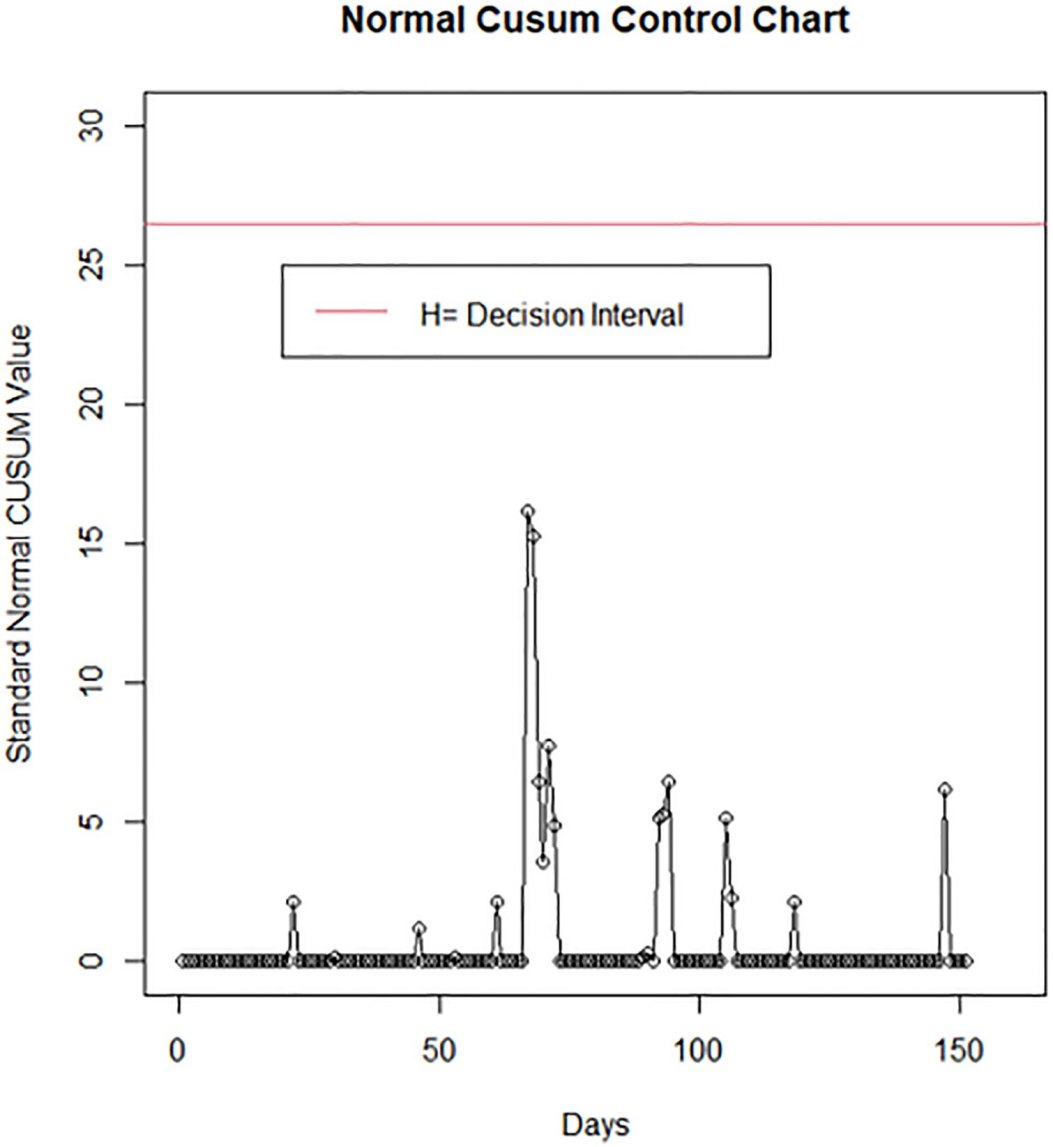
Standard normal-CUSUM control chart of COVID-19 number of deaths in Nigeria.

Furthermore, the performance of the standard normal-CUSUM control chart is evaluated assuming that the COVID-19 data follow the normal distribution for the period of study and is compared with the proposed Gamma-CUSUM chart. A plot of the standard normal-CUSUM control chart statistics in Table 3 when the COVID-19 data follow the normal distribution is presented in Fig 4.

**Table 3:** Upper Normal- CUSUM statistic for COVID-19 number of deaths.

Day	Number of Deaths	Upper CUSUM Statistic, $C_{n}^{+}$	Day	Number of Deaths	Upper CUSUM Statistic $C_{n}^{+}$	Day	Number of Deaths	Upper CUSUM Statistic $C_{n}^{+}$
1	3	0	52	12	0	103	4	0
2	0	0	53	15	0.141227	104	8	0
3	0	0	54	1	0	105	20	5.14123
4	1	0	55	8	0	106	12	2.28245
5	1	0	56	10	0	107	11	0
6	1	0	57	9	0	108	2	0
7	4	0	58	12	0	109	2	0
8	2	0	59	7	0	110	8	0
9	2	0	60	4	0	111	5	0
10	1	0	61	17	2.14123	112	5	0
11	3	0	62	5	0	113	1	0
12	3	0	63	12	0	114	4	0
13	3	0	64	8	0	115	5	0
14	1	0	65	13	0	116	8	0
15	3	0	66	4	0	117	14	0
16	5	0	67	31	16.1412	118	17	2.14123
17	0	0	68	14	15.2825	119	3	0
18	4	0	69	6	6.42368	120	6	0
19	7	0	70	12	3.56491	121	6	0
20	7	0	71	19	7.70614	122	3	0
21	10	0	72	12	4.84736	123	5	0
22	17	2.14123	73	7	0	124	6	0
23	2	0	74	8	0	125	0	0
24	6	0	75	9	0	126	10	0
25	5	0	76	7	0	127	7	0
26	5	0	77	5	0	128	1	0
27	4	0	78	4	0	129	1	0
28	10	0	79	7	0	130	2	0
29	11	0	80	8	0	131	4	0
30	15	0.141227	81	1	0	132	4	0
31	7	0	82	**7**	0	133	7	0
32	8	0	83	13	0	134	4	0
33	6	0	84	13	0	135	1	0
34	3	0	85	12	0	136	5	0
35	4	0	86	6	0	137	2	0
36	5	0	87	11	0	138	3	0
37	6	0	88	9	0	139	3	0
38	9	0	89	15	0.141227	140	1	0
39	1	0	90	15	0.282454	141	0	0
40	8	0	91	5	0	142	0	0
41	11	0	92	20	5.14123	143	2	0
42	10	0	93	15	5.28245	144	0	0
43	0	0	94	16	6.42368	145	10	0
44	5	0	95	4	0	146	4	0
45	7	0	96	10	0	147	21	6.14123
46	16	1.14123	97	6	0	148	3	0
47	5	0	98	9	0	149	3	0
48	5	0	99	3	0	150	3	0
49	2	0	100	6	0	151	4	0
50	12	0	101	11	0			
51	14	0	102	12	0			

A comparison of the plots in Figs 3 and 4 revealed that the Gamma-CUSUM detect out-of-control signals on day 68 whereas the standard normal-CUSUM chart didn’t signal an out-of-control. This establishes the adequacy and efficiency of the Gamma-CUSUM chart for the monitoring of COVID -19 number of deaths.

## Conclusion

In this study, the reported COVID-19 number of deaths was modeled using the generalized gamma family of distributions. The best-fit distribution was obtained as the gamma distribution for the COVID-19 number of deaths extracted from the NCDC website for the period 11^th^ April to 7^th^ September 2020. Thereafter, a Gamma-CUSUM control chart was developed for monitoring the COVID-19 number of deaths in Nigeria. The results show that the COVID-19 number of deaths consistently rises between 68 days to 120 days, indicating that the COVID-19 number of deaths within the period was beyond the control limits and requires further investigations to ascertain the problem therein. Also, the performance of the Gamma-CUSUM chart was compared with the standard normal-CUSUM chart and the results revealed the superiority of the Gamma-CUSUM chart. The results showed that the distributional knowledge of infectious diseases is essential for efficient monitoring of infections and deaths arising from the infections. The proposed study can be extended for neutrosophic statistics as future research work when the data is from an uncertain environment. Similarly, the CUSUM chart for the generalized gamma distribution to monitor time-between-COVID 19 deaths is another future research work worth investigating.

## Supporting information

S1 Table COVID-19 data on number of infected persons and deaths. (DOCX)
