# Supplementary material for: Generalized Gamma-CUSUM control chart with application of COVID-19 deaths
Source: PLoS One. 2023 Feb 2;18(2):e0281360. doi: 10.1371/journal.pone.0281360 (PMC9894481; doi:10.1371/journal.pone.0281360)
Supplement: S1 Table — (DOCX) [file pone.0281360.s001.docx]

**S1 Data. COVID-19 Data on Number of Infected Persons and Deaths**

| **Date** | **Number of Infected Person** | **Number of Deaths** | **Date** | **Number of Infected Person** | **Number of Deaths** | **Date** | **Number of Infected Person** | **Number of Deaths** |
| --- | --- | --- | --- | --- | --- | --- | --- | --- |
| April 11 | 13 | 3 | 31 | 307 | 14 | 20 | 562 | 11 |
| 12 | 5 | 0 | June 1 | 416 | 12 | 21 | 576 | 12 |
| 13 | 20 | 0 | 2 | 241 | 15 | 22 | 543 | 4 |
| 14 | 30 | 1 | 3 | 347 | 1 | 23 | 604 | 8 |
| 15 | 34 | 1 | 4 | 350 | 8 | 24 | 591 | 20 |
| 16 | 35 | 1 | 5 | 328 | 10 | 25 | 439 | 12 |
| 17 | 57 | 4 | 6 | 389 | 9 | 26 | 555 | 11 |
| 18 | 49 | 2 | 7 | 253 | 12 | 27 | 648 | 2 |
| 19 | 85 | 2 | 8 | 315 | 7 | 28 | 624 | 2 |
| 20 | 38 | 1 | 9 | 663 | 4 | 29 | 404 | 8 |
| 21 | 0 | 3 | 10 | 409 | 17 | 30 | 481 | 5 |
| 22 | 208 | 3 | 11 | 681 | 5 | 31 | 462 | 5 |
| 23 | 108 | 3 | 12 | 627 | 12 | August 1 | 386 | 1 |
| 24 | 114 | 1 | 13 | 501 | 8 | 2 | 304 | 4 |
| 25 | 87 | 3 | 14 | 403 | 13 | 3 | 288 | 5 |
| 26 | 91 | 5 | 15 | 573 | 4 | 4 | 304 | 8 |
| 27 | 64 | 0 | 16 | 490 | 31 | 5 | 457 | 14 |
| 28 | 195 | 4 | 17 | 587 | 14 | 6 | 354 | 17 |
| 29 | 196 | 7 | 18 | 745 | 6 | 7 | 443 | 3 |
| 30 | 204 | 7 | 19 | 667 | 12 | 8 | 453 | 6 |
| May 1 | 238 | 10 | 20 | 661 | 19 | 9 | 437 | 6 |
| 2 | 218 | 17 | 21 | 436 | 12 | 10 | 290 | 3 |
| 3 | 170 | 2 | 22 | 675 | 7 | 11 | 423 | 5 |
| 4 | 244 | 6 | 23 | 452 | 8 | 12 | 453 | 6 |
| 5 | 148 | 5 | 24 | 649 | 9 | 13 | 373 | 0 |
| 6 | 195 | 5 | 25 | 594 | 7 | 14 | 329 | 10 |
| 7 | 381 | 4 | 26 | 684 | 5 | 15 | 325 | 7 |
| 8 | 386 | 10 | 27 | 779 | 4 | 16 | 298 | 1 |
| 9 | 239 | 11 | 28 | 490 | 7 | 17 | 417 | 1 |
| 10 | 248 | 15 | 29 | 566 | 8 | 18 | 410 | 2 |
| 11 | 242 | 7 | 30 | 561 | 1 | 19 | 593 | 4 |
| 12 | 146 | 8 | July 1 | **790** | **7** | 20 | 476 | 4 |
| 13 | 184 | 6 | 2 | 626 | 13 | 21 | 340 | 7 |
| 14 | 191 | 3 | 3 | 454 | 13 | 22 | 601 | 4 |
| 15 | 288 | 4 | 4 | 603 | 12 | 23 | 322 | 1 |
| 16 | 171 | 5 | 5 | 544 | 6 | 24 | 321 | 5 |
| 17 | 338 | 6 | 6 | 575 | 11 | 25 | 252 | 2 |
| 18 | 216 | 9 | 7 | 503 | 9 | 26 | 221 | 3 |
| 19 | 226 | 1 | 8 | 460 | 15 | 27 | 296 | 3 |
| 20 | 276 | 8 | 9 | 499 | 15 | 28 | 160 | 1 |
| 21 | 339 | 11 | 10 | 575 | 5 | 29 | 250 | 0 |
| 22 | 245 | 10 | 11 | 664 | 20 | 30 | 138 | 0 |
| 23 | 265 | 0 | 12 | 571 | 15 | 31 | 143 | 2 |
| 24 | 313 | 5 | 13 | 595 | 16 | September 1 | 239 | 0 |
| 25 | 229 | 7 | 14 | 463 | 4 | 2 | 216 | 10 |
| 26 | 276 | 16 | 15 | 643 | 10 | 3 | 125 | 4 |
| 27 | 389 | 5 | 16 | 595 | 6 | 4 | 155 | 21 |
| 28 | 182 | 5 | 17 | 600 | 9 | 5 | 162 | 3 |
| 29 | 387 | 2 | 18 | 653 | 3 | 6 | 100 | 3 |
| 30 | 553 | 12 | 19 | 556 | 6 | 7 | 155 | 3 |
|  |  |  |  |  |  | 8 | 29 | 4 |
